# The effect of switch therapy to tenofovir versus entecavir maintenance on recurrence of hepatocellular carcinoma after surgery (SWITE): study protocol for a randomized controlled trial

**DOI:** 10.1186/s13063-023-07822-y

**Published:** 2023-12-02

**Authors:** Wei Peng, Mengshi Yi, Xin Qi, Weili Qi, Chuan Li, Tianfu Wen

**Affiliations:** 1 Division of Liver Surgery, Department of General Surgery, West China Hospital, Sichuan University, Chengdu, 610041 China; 2 Chinese Evidence-based Medicine Center, West China Hospital, Sichuan University, Chengdu, China; 3 Deparment of Hepatobiliary Surgery, the First Affiliated Hospital of Army Medical University, Chongqing, China; 4 West China School of Medicine, Sichuan University, Chengdu, China

**Keywords:** Antiviral therapy, Entecavir, Hepatocellular carcinoma, Recurrence, Tenofovir disoproxil fumarate

## Abstract

**Background:**

Antiviral therapy has been reported to be associated with lower recurrence rate of hepatocellular carcinoma (HCC) for patients with hepatitis B virus (HBV) infection. While entecavir (ETV) and tenofovir disoproxil fumarate (TDF) were both recommended as first-line therapies for HBV patients, recent retrospective studies proposed a lower incidence rate of HCC occurrence or recurrence in those receiving TDF compared ETV. However, the survival benefits of switching to TDF therapy after prolonged ETV treatment before surgery remain uncertain. We delineate the rationale and design of SWITE, a randomized, open-label, phase III trial contrasting TDF switch therapy versus ETV maintenance in HBV-related HCC patients.

**Methods and analysis:**

This is a prospective, randomized, controlled, single-center study with two parallel groups of patients with HBV-related HCC who have received long-term ETV therapy before surgery. West China Hospital will enroll 238 patients, randomized in a 1:1 ratio to TDF switch therapy or ETV maintenance after surgery. The primary endpoint of this study is 3-year recurrence free survival (RFS), with the secondary endpoint being 3-year overall survival (OS) after curative surgery of HCC. Safety events will be diligently recorded.

**Ethics and dissemination:**

The study protocol aligns with the ethical guidelines of the 1975 Declaration of Helsinki. It was approved by ethics committee of West China Hospital (approval number: 2022-074) and was registered with chictr.org.cn (chiCTR2200057867). Informed consent will be obtained from all participants. The results of this trial will be published in peer-reviewed journals and presentations at national and international conferences relevant to this topic.

**Trial registration:**

chiCTR2200057867. Date of registration is March 20 2022.

## Introduction

Hepatocellular carcinoma (HCC) stands as a prevalent malignancy globally [1]. In China, the elevated crude mortality rate of HCC ranks it as the second leading cause of cancer-related death nationally, primarily attributed to endemic chronic hepatitis B virus infection (CHB) [2]. Between 2010 and 2014, the 5-year relative survival rate for Chinese HCC patients was a mere 14.1% [3]. Despite liver resection being the primary treatment for HCC, particularly for those in the early stages with well-preserved liver function, the clinical outcome remains unsatisfactory due to a high recurrence rate. Even in the early stages of HCC, the postoperative 5-year recurrence rate can range from 50 to 70% [4, 5]. Prognostic factors such as tumor size, microvascular invasion, liver cirrhosis, alpha-fetoprotein (AFP) levels, and the viral replication status of HBV influence HCC prognosis [6], with viral load being the most clinically manageable.

Numerous studies have reported that antiviral therapy is associated with a lower HCC recurrence rate after surgical resection [7–9]. For instance, a national cohort study of 4569 patients comparing those with antiviral therapy and untreated patients with resected HBV-related HCC found antiviral therapy to be an independent predictor for lower HCC recurrence [10]. Even in a randomized controlled trial involving patients with low HBV DNA levels, antiviral therapy significantly correlated with improved overall survival (OS) and recurrence-free survival (RFS) [11].

Entecavir (ETV) and tenofovir disoproxil fumarate (TDF) are recommended as first-line therapies for suppressing viral replication [12, 13], demonstrating long-term efficacy in reducing HCC risk for CHB patients [14, 15]. Recent studies on ETV and TDF for preventing recurrence after curative resection in HBV-related HCC consistently favor TDF [16, 17], aligning with our previous findings [18–20]. However, in the majority of these studies, antiviral therapy was initiated only after HCC diagnosis, as the patients had not been diagnosed with HBV infection before. Whether ETV or TDF treatment after curative resection results in different prognostic outcomes in CHB patients who received long-term ETV treatment prior to HCC development remains unclear. Additionally, these studies were prone to bias due to their retrospective nature. Hence, we aim to conduct a prospective randomized controlled study (SWITE) in a single center to directly investigate the effect and safety of switching from ETV to TDF for patients with prolonged ETV therapy before surgery.

## Methods and analysis

### Aims of the study

The study aims to (1) directly compare the efficacy of maintaining ETV and switching to TDF on HCC recurrence risk and survival in patients with prolonged ETV therapy before surgery, (2) compare the safety of ETV maintenance and TDF switch after surgery through adverse event records, and (3) offer evidence and guidance for perioperative antivirus strategies in HBV-related HCC patients with prolonged ETV therapy.

### Study design

**Fig. 1:**
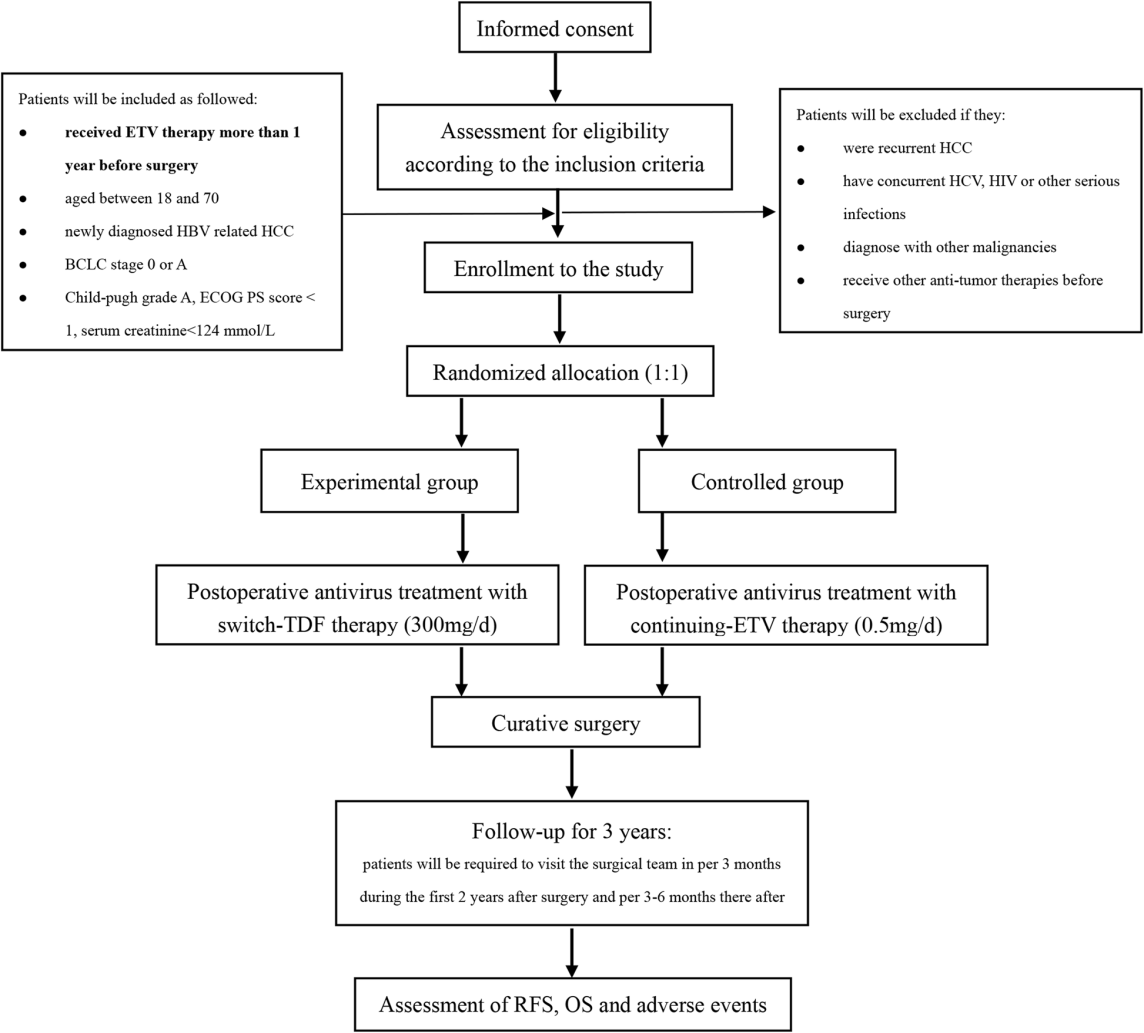
Flow chart of the study design

This trial adopts a prospective, randomized, controlled, single-center design and is scheduled to commence at West China Hospital from May 2022 to August 2027. Figure 1 illustrates the study process.

### Trial registration

This study was registered with chictr.org.cn (chiCTR2200057867).

### Participant recruitment

From May 2022 to May 2024, patients with HBV-related HCC who have received long-term ETV therapy before surgery that scheduled for hepatectomy in West China Hospital will be screened for eligibility for this trial.

### Inclusion criteria

The inclusion criteria are as follows: (1) aged 18 to 70; (2) newly diagnosed with HBV-related HCC following clinical diagnosis criteria of the American Association for the Study of Liver Diseases (AASLD) [21]; (3) Barcelona Clinic Liver Cancer (BCLC) stage 0 or A, receiving curative liver resection or ablation; (4) ETV monotherapy for over 1 year with pre-surgery HBV-DNA load under 100 IU/mL; (5) Child-Pugh class A with or without cirrhosis, Eastern Cooperative Oncology Group (ECOG) performance status (PS) score ≤ 1; (6) appropriate renal function (serum creatinine < 124 mmol/L).

### Exclusion criteria

The exclusion are as follows: (1) recurrent HCC; (2) concurrent HCV, HIV, or other serious infections; (3) other malignancies or mental illness; (4) prior anti-tumor therapies pre-surgery, including portal vein ligation/embolization, radiofrequency ablation, transarterial chemoembolization, etc.; (5) participation in conflicting ongoing trials.

Participants can withdraw at any time, and poor compliance may lead to removal. Withdrawal reasons will be recorded in case report forms (CRFs), and data will be analyzed following the intention-to-treat principle [22].

### Sample size calculation

The sample size calculation was performed using the software PASS (version 2015). The parameters were obtained from our previous randomized clinical trial, which evaluated the efficacy of TDF and ETV in early-stage HBV-related HCC patients after surgery. In that study, the 3-year RFS rates were 70.4% and 82.4% for patients receiving ETV and TDF, respectively. Assuming 3-year RFS rates of 70% for the control group and 85% for the treatment group, with a one-sided alpha of 0.05 and 80% power, the corresponding sample size is 190. Accounting for a 20% dropout rate, a total of 238 patients will be included in this study.

### Randomization and allocation

Block randomization with a block length of four will be employed. For each block, enrolled participants will undergo 1:1 randomization into the continuing-ETV therapy group and the switch-TDF therapy group. Randomization numbers will be generated using STATA/SE 15.1 and sealed in envelopes.

### Intervention

Participants will be equally randomized into two groups: the experimental group (postoperative antivirus strategies with switch-TDF therapy) and the controlled group (postoperative antivirus strategies with continuing-ETV therapy). TDF dosage is 300 mg per day, and ETV dosage is 0.5 mg per day.

### Follow-up

Patient follow-up begins post-hospital discharge. Visits to the surgical team will occur every 3 months during the first 3 years post-surgery. Check-ups, including CT and/or MRI, ultrasound, routine blood tests, liver and kidney function assessments, AFP, PIVKA, and HBV DNA level measurements, will be conducted at each visit (Table 1).

**Table 1:** Participant timeline

	Study period												
	Enrollment/allocation	Post-allocation											Close-out
Timepoint	Pre-allocation	0 months	1 month	3 months	6 months	9 months	12 months	15 months	18 months	21 months	24 months	30 months	36 months
**Informed consent:**	×												
**Eligibility screen**	×												
**CT or MRI**	×												
**Laboratory test**	×												
**Allocation**	×												
**Surgery**		×											
**Interventions**													
**Post-operative switch-TDF therapy**		×	×	×	×	×	×	×	×	×	×	×	×
**Post-operative continuing-ETV therapy**		×	×	×	×	×	×	×	×	×	×	×	×
**Assessments**													
**First CT after surgery**			×										
**Regular follow-up (lab, CT or MRI)**				×	×	×	×	×	×	×	×	×	×
**Adverse events**		×	×	×	×	×	×	×	×	×	×	×	×

### Endpoints

The primary endpoint is 3-year RFS, defined as the interval from the operation to the date of the first diagnosed HCC recurrence, recorded in months. The secondary endpoint is 3-year OS, defined as the interval from surgery to death or the date of the last follow-up, recorded in months.

### Safety reporting

Both treatments’ safety will be evaluated through vital signs, physical exams, laboratory data, adverse events (AEs), and serious adverse events (SAEs). AEs encompass unexpected medical events or worsening conditions not necessarily linked to treatment, including hypophosphatemia, dizziness, diarrhea, nausea, vomiting, rash, fatigue, flatulence, and transaminase elevation. SAEs are events leading to death, life-threatening situations, hospitalization, deformities, birth defects, or significant permanent damages. AEs and SAEs will be documented in CRFs, with SAEs reported to ethics and administrative departments within 24 h.

Definition of the end of study is as follows: (1) participants have completed the last follow-up, (2) participants exhibit recurrence via imaging tests, and (3) participants die from any cause.

### Mid-term analysis

Besides final analysis, a mid-term analysis will be scheduled in the study. The purpose of mid-term analysis is about the safety and efficacy of the study. The scheduled time for mid-term analysis is set at 1 year after the last enrolled patient. If the RFS invalid hypothesis is rejected prior to final analysis, the trial might be discontinued.

### Data collection and management

Investigators and coordinators will receive specialized training for data collection. All trial-related data will be recorded on study-specific CRFs. In particular, plenty factors, including different stages of cirrhosis, would play an important role in survival of patients with HCC after surgery. Comprehensive clinical data, including Ishak score of the resected specimen, fibrosis-4 index, aminotransferase-to-platelet ratio index, and other parameters for liver cirrhosis to evaluate the baseline stage of cirrhosis, will be prospectively collected. Besides, the death from decompensation of cirrhosis, HCC recurrence, and other reasons will be precisely recorded and recognized during the follow-up since decompensation in patients with cirrhosis is a competing event from HCC recurrence. Baseline assessments will be conducted pre-randomization, and missing data will be stored until received or confirmed as unavailable. Personal information will remain confidential. Data will be entered into Excel by two independent collectors, using participant numbers for identification. Access to information will be restricted. A data manager will oversee routine accuracy checks and address any issues with the investigators. Hard copy documents will be stored securely, and electronic files will have password protection. All documents will be saved for at least 5 years post-publication, with original data available upon request to the principal investigator.

### Statistical analysis

Background measures will be presented as mean ± standard deviation for continuous variables or as numbers/percentages for categorical variables. Continuous variables will be compared using a two-sided student *t*-test or Mann-Whitney *U* test, while categorical variables will undergo the chi-square test or Fisher’s exact test. Patient survival (RFS and OS) will be calculated via the Kaplan-Meier method with log-rank test. In particular, cancer-specific survival will also be calculated since decompensation serves as a competing event from HCC recurrence for death. Stage of cirrhosis, tumor burden, antiviral regimen, and other factors which may affect survival after surgery would be considered when conducting survival analysis. Generalized linear models will analyze primary and secondary endpoints. An ITT analysis, including protocol deviations, will be conducted using IBM SPSS version 26.0 software. A *p*-value < 0.05 indicates statistical significance.

## Ethics and dissemination

### Ethics approval

The study protocol was approved by the Ethics Committee of West China Hospital, Sichuan University.

### Informed consent

Trained researchers will provide detailed study information, including design, aspects, benefits, and potential harms, to potential participants before enrollment. Enrolled patients will sign written informed consent, ensuring confidentiality of personal information.

### Dissemination

Study findings will be disseminated through national and international conferences as well as peer-reviewed publications.

### Patient and public involvement

No patient is involved.

## Discussion

In the case of most hepatocellular carcinoma (HCC) patients, surgical resection or liver transplantation remains the sole curative modalities. However, the recurrence rate of HCC can ascend to 41–50% within the initial 24 months post-surgical resection (early recurrence) and up to 20% beyond the 2-year mark (late recurrence) [23]. Studies indicate that an elevated viral load is correlated with a heightened HCC recurrence rate and diminished survival post-surgery [4, 24]. Plausible mechanisms suggest that the heightened viral load and hepatic inflammatory activity may contribute to necrosis, ensuing regeneration of residual hepatocytes, inducing DNA mutations, instability, and ultimately fostering HCC recurrence [16]. A mounting body of evidence supporting a favorable prognosis for resected HBV-related HCC with antiviral therapy has prompted international and local clinical practice guidelines to advocate for antiviral intervention pre- and/or post-surgical resection in patients with HBV-related HCC to forestall disease progression and mitigate HCC recurrence [12, 23].

The foremost antiviral agents, namely ETV and TDF, serve as first-line therapies. ETV, a cyclopentyl guanosine analogue, has the potential to inhibit HBV polymerase, achieving a mean 6.9 log decline of serum HBV DNA. Previous investigations reveal a cumulative probability of undetectable HBV DNA at 94–97% with 5-year ETV treatment [25, 26] and 83–90% with 3-year ETV treatment [27–29] in HBeAg-positive patients. TDF, an acyclic adenine nucleotide analogue effective against both HBV and HIV, demonstrates a 3-year cumulative virologic suppression rate of 93.3% in an Asian cohort [30]. Another study reports a 7-year TDF treatment achieving undetectable HBV DNA in 99.3% of HBV patients [31].

As established earlier, antiviral therapy is linked to a reduced HCC recurrence rate post-surgical resection. Current evidence leans towards TDF as a superior choice over ETV in improving HCC prognosis after curative surgery. The potential mechanism behind the lower HCC recurrence rate with TDF therapy, as opposed to ETV therapy, may be attributed to higher serum interferon (IFN)-λ3 levels induced by TDF, but not ETV [32]. IFN-λ has demonstrated antitumor activity in murine HCC models [33, 34], potentially accounting for the divergent recurrence rates observed in patients treated with TDF versus ETV. In a separate in vitro study, TDF pretreatment, unlike ETV, inhibited enteric lipopolysaccharide-mediated production of interleukin (IL)-10 [35], while inducing IL-12. IL-10, an inhibitor of CD8+ T cells, is suppressed, and IL-12 directly stimulates T cells and natural killer (NK) cells. Consequently, TDF therapy may restore the functionality of T cells and NK cells through down-regulating IL-10 and up-regulating IL-12 [16].

Despite prior studies highlighting a superior prognosis with TDF treatment post-surgical resection compared to ETV treatment, these investigations were retrospective and failed to address the necessity of transitioning to TDF therapy after prolonged ETV use. Notably, in China, a nation with a high prevalence of CHB, ETV remains the predominant antiviral drug, constituting nearly 80% of antiviral agents purchased by patients [36]. In clinical practice, many HBV-related HCC patients undergo prolonged ETV therapy before an HCC diagnosis, with TDF potentially overlooked due to economic constraints and concerns about TDF-related renal dysfunction and decreased bone density. Consequently, this study aims to ascertain whether transitioning to TDF treatment post-hepatectomy provides benefits for patients who have received prolonged ETV therapy before surgery. We anticipate that the findings of this study will furnish valuable clinical insights for future treatments.

## Data Availability

Data sharing is not applicable to this article, as no datasets were generated or analyzed during the current study.

## Abbreviations

HCC: Hepatocellular carcinoma
ETV: Entecavir
TDF: Tenofovir disoproxil fumarate
RFS: Recurrence-free survival
OS: Overall survival
CHB: Chronic hepatitis B virus infection
CRF: Case report forms
AEs: Adverse events (AEs)
SAEs: Serious adverse events
IFN: Interferon
IL: Interleukin
NK: Natural killer cells
